# Iatrogenic pneumothorax after breast reduction surgery caused by local anesthesia infiltration – a case report

**DOI:** 10.1080/23320885.2022.2043753

**Published:** 2022-02-23

**Authors:** Marko T. Ristola, Ilkka Koskivuo, Salvatore Giordano

**Affiliations:** Department of Plastic Surgery, Turku University Hospital, Turku, Finland

**Keywords:** Case report, pneumothorax, mammoplasty, local anesthesia, complication, postoperative

## Abstract

We present a case of a 44-year-old woman, who underwent bilateral breast reduction mammoplasty and suffered a unilateral pneumothorax that was detected postoperatively. Infiltration of a local anesthetic was considered the cause of the pneumothorax. We recommend a more tangential direction of needle placement when infiltrating a local anesthetic.

## Introduction

Although some authors have performed breast reduction under intravenous sedation and local anesthesia [1], a more common technique is to perform the operation under general anesthesia, and simply infiltrate the local anesthetic intradermally for its hemostatic properties, especially to perform de-epithelialization of the pedicle in a more bloodless manner [2]. In our department, local anesthesia infiltration is performed more for its hemostatic properties and postoperative pain management.

Iatrogenic pneumothorax due to local anesthesia infiltration is an extremely rare complication in breast surgery. There are some reports of iatrogenic pneumothorax after breast augmentation surgery, that have speculated the cause to be injecting local anesthetic [3–5], and a survey in 2005 speculated that the incidence of this complication may be underestimated [6]. However, the authors found only one previous reported case of pneumothorax thought to be caused by infiltrating a local anesthetic in breast reduction surgery [7]. While smaller pneumothoraces may be treated conservatively, more significant cases often require drainage with either a minimally invasive chest drainage kit, or a more invasive open surgical chest drainage tube.

## Case report

The patient was a 44-year-old Caucasian female, who had previously undergone endovascular surgery for two aneurysms of the right middle cerebral artery, abdominoplasty and plication of rectus abdominis diastasis, and blepharoplasty for dermatochalasis. She had no regular medication and no known allergies. She had a history of smoking, but had quit some years earlier. She had a normal BMI of 24.6 kg/m^2^.

**Figure 1.**
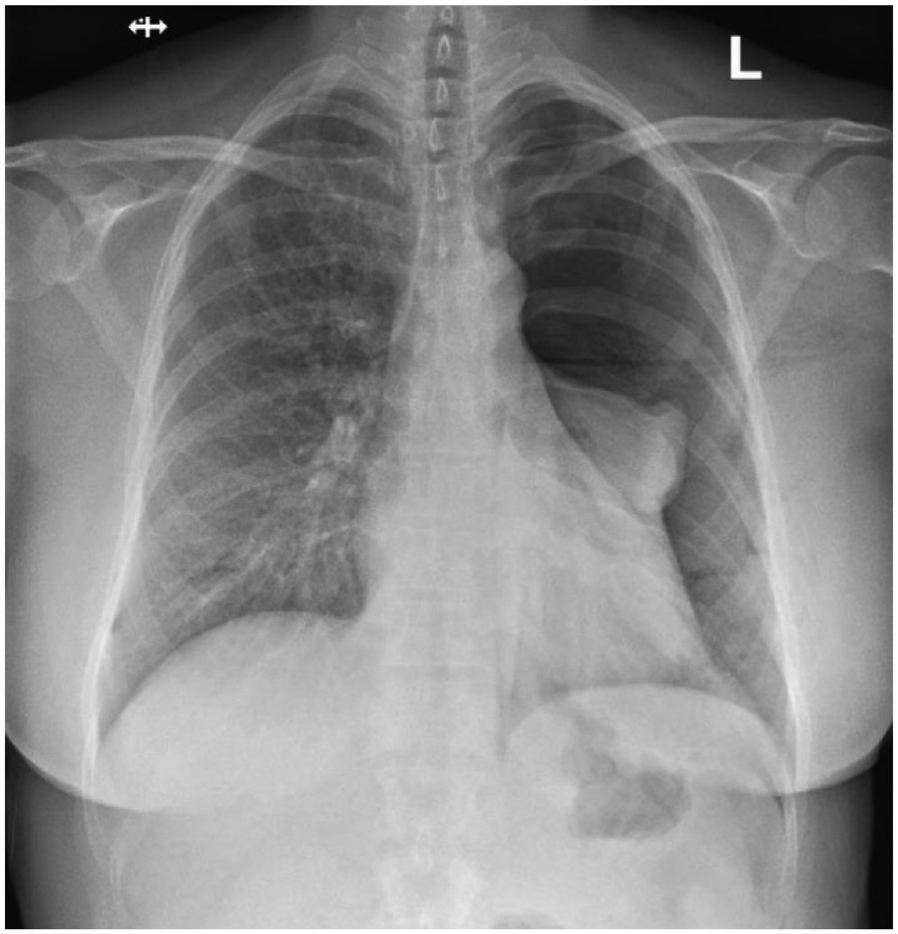
Postoperative chest X-ray image showing pneumothorax on the left side.

On 12 September 2019 she underwent breast reduction mammoplasty for breast hyperplasia and subsequent neck and shoulder region pain and stiffness. The mammoplasty was performed under general anesthesia with propofol and an I-Gel® laryngeal mask with sevoflurane. Before the initial incision, one of the operating surgeons, a senior consultant plastic surgeon and chief of the department of plastic surgery, infiltrated a local anesthetic consisting of 20 ml of ropivacaine 7.5 mg/ml, 20 ml of lidocaine 5 mg/ml with adrenalin 10 microg/ml in each breast, for a total of 40 ml of local anesthetic solution per breast. The needle used was a 21 G needle, 0.8 mm thick and 80 mm long. The infiltration technique used was a combination of intradermal and intramammary injection deep into the breast tissue, in a somewhat perpendicular direction to the chest wall. After infiltration of the local anesthetic solution, the procedure was performed in a standard fashion using a Wise pattern technique with a superomedial pedicle. During the operation, no inconsistencies or abnormalities were noted in either the surgical side or the anesthesia side, and the operation was carried out routinely.

**Figure 2.**
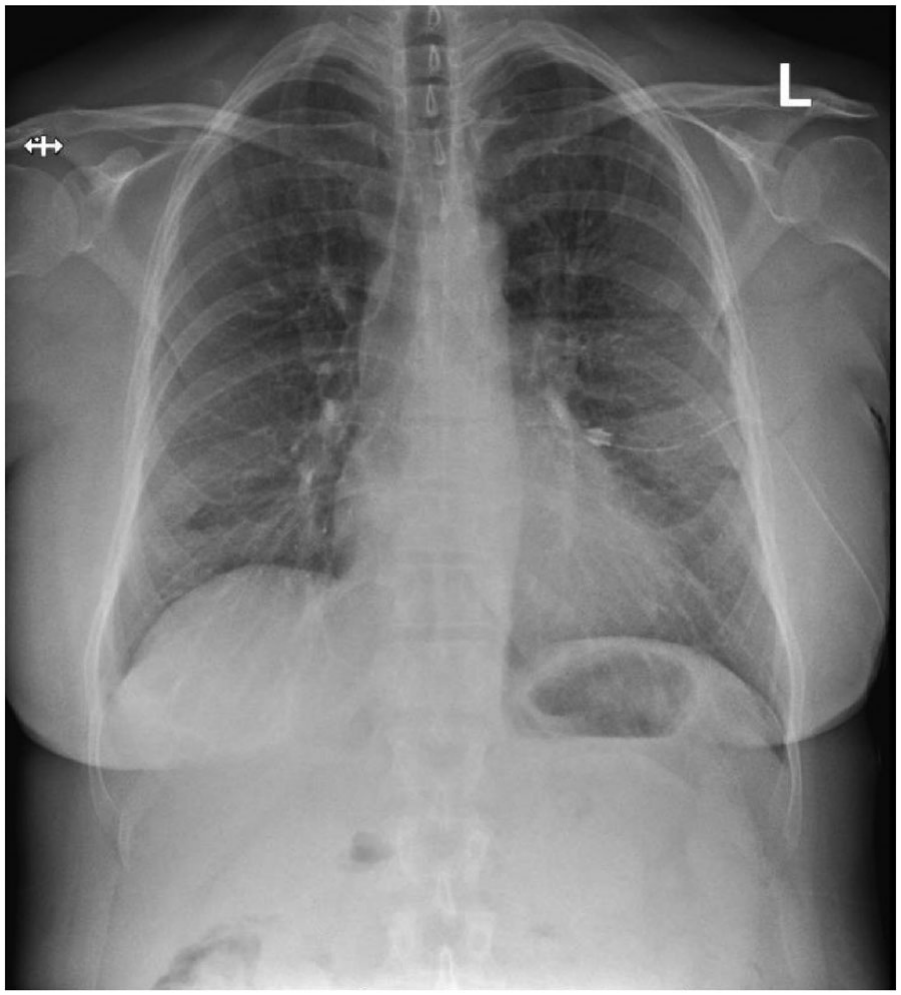
Chest X-ray after insertion of a chest drainage tube showing resolution of the pneumothorax.

After the operation, the patient came out of the anesthesia without any trouble, and was transferred to the recovery room. The patient was noted to be breathing without trouble, with an oxygen saturation of over 95%. After a few hours of monitoring, the patient was feeling well and was discharged. The following day, the patient called the department of plastic surgery and reported that she had started suffering from shortness of breath and a sharp pain in the left clavicular area after arriving home on the day of the operation. A nurse received the call and advised the patient to remove her supportive bandages. When this did not alleviate her symptoms, the patient went into her local hospital emergency room, as advised by the nurse during the phone call.

Upon arrival at the District Hospital of Salo emergency room, the patient’s vital signs, basic bloodwork, and ECG were taken. The ECG, most of the bloodwork, and other vital signs were found normal, but her oxygen saturation was slightly below normal at 93 percent, and blood leukocyte count was elevated at 15.5 × 10^9^/l. A chest radiograph revealed a large pneumothorax on the left side, as well as slight subcutaneous emphysema (Figure 1). After a failed attempt at pleural drainage with a Seldinger® chest drainage kit, a 20 Fr chest drainage tube was inserted using an open, surgical approach (Figure 2). The patient was admitted to the surgical ward in the District Hospital of Salo, and spent three days there with the chest drain in place. After three days, a new chest radiograph was taken and found normal. The chest drain was then removed, and the patient was discharged. After that, the patient did not experience any significant pain or trouble breathing anymore, and recovered completely from the pneumothorax. No further follow-up imaging was performed. The patient developed no long-term morbidity due to the pneumothorax.

## Discussion

In a series of 1322 procedures on 1152 patients, iatrogenic pneumothorax as a result of thoracic paravertebral blockade was described in 0.26%. The article describes using a single-shot T2/3 technique to achieve analgesia for both the breast and the axilla. The surgical procedures included wide local excision and sentinel node biopsy (495 patients), wide local excision (265 patients), mastectomy and axillary clearance (82 patients), mastectomy and sentinel node biopsy (72 patients), bilateral mastectomy (49 patients), axillary clearance (42 patients), wide local excision and axillary clearance (40 patients), bilateral wide local excision (27 patients), change of implants (27 patients), sentinel node biopsy (23 patients), bilateral reduction (17 patients), unilateral reduction (14 patients), and mastectomy and reconstruction (9 patients). Of these patients, a postoperative chest radiograph was taken in 37 patients (3.2%), mainly due to desaturation, chest pain, or suspicion of pleural puncture during the blockade procedure. All of the patients in this series could be managed conservatively without the need for pleural drainage [8]. Similarly, we do not recommend to perform chest radiographs routinely after breast reduction surgery without clinical indications.

Local anesthesia infiltration has been considered to be the cause of intraoperative pneumothorax in some patients undergoing breast augmentation in [3–5]. In our case, as well, we found the most likely cause of pneumothorax to be intrapleural puncture with local anesthesia infiltration needle. This complication could have possibly been avoided with more tangentially directed infiltration. We therefore recommend, that if infiltration of local anesthesia into the deeper tissue of the breast is performed, the direction should be more tangential than perpendicular to the chest wall, and extreme caution should be practiced.

## Informed consent

Before submitting this paper, the group acquired informed consent from the patient. Ten months following the pneumothorax, she herself experienced no long-term morbidity due to the pneumothorax.

## References

[1] Zukowski ML, Ash K, Klink B, et al. Breast reduction under intravenous sedation: a review of 50 cases. Plast Reconstr Surg. 1996;97(5):952–956. 8618998 10.1097/00006534-199604001-00010

[2] Bennett KG, Gilman RH. Intradermal infiltration of local anesthetic-rapid and bloodless deepithelialization of the breast pedicle. Plast Reconstr Surg Glob Open. 2017;5(2):e1225. 28280667 10.1097/GOX.0000000000001225 PMC5340482

[3] Pfulg M, Favre S, Verdeja R. Bilateral pneumothoraces: a rarely described complication following augmentation mammaplasty. Aesthet Surg J. 2005;25(1):49–52. 19338788 10.1016/j.asj.2004.11.004

[4] Averick R, Hahn B. Pericardial effusion and pneumothorax after breast augmentation. J Emerg Med. 2011;41(1):79–81. 19217239 10.1016/j.jemermed.2008.09.025

[5] Kaye AD, Eaton WM, Jahr JS, et al. Local anesthesia infiltration as a cause of intraoperative tension pneumothorax in a young healthy woman undergoing breast augmentation with general anesthesia. J Clin Anesth. 1995;7(5):422–424. 7576680 10.1016/0952-8180(95)00059-q

[6] Osborn J, Stevenson TR. Pneumothorax as a complication of breast augmentation. Plast Reconstr Surg. 2005;116(4):1122–1126. 16163105 10.1097/01.prs.0000179182.58036.a7

[7] Mavridis S, Gnauk HG, Schumacher M, et al. Bilateral pneumothoraces complicating reduction mammoplasty: a case report. BMC Surg. 2013;13(1):29. 23890488 10.1186/1471-2482-13-29 PMC3728039

[8] Kelly ME, Mc Nicholas D, Killen J, et al. Thoracic paravertebral blockade in breast surgery: is pneumothorax an appreciable concern? A review of over 1000 cases. Breast J. 2018;24(1):23–27. 28557058 10.1111/tbj.12831

